# (*E*)-4-Amino-*N*′-(3-nitro­benzyl­idene)benzohydrazide

**DOI:** 10.1107/S1600536812014110

**Published:** 2012-04-06

**Authors:** Shu-Qing Xu

**Affiliations:** a School of Ocean, Qinzhou University, Qinzhou, Guangxi 535000, People’s Republic of China

## Abstract

In the title compound, C_14_H_12_N_4_O_3_, the dihedral angle between the benzene rings is 7.6 (4)°. In the crystal, infinite sheets linked by N—H⋯O and bifurcated N—H⋯(O,N) hydrogen bonds propagate in the (10-1) plane, in which *R*_4_^4^(36) loops are apparent. Neighbouring layers may inter­act by way of very weak π–π stacking inter­actions [centroid–centroid distances = 3.9329 (13) and 4.0702 (13) Å].

## Related literature

For related structures and background references to hydrazones, see: Cao (2009 ▶); Zhou & Yang (2010 ▶). For graph-set notation, see: Bernstein *et al.* (1995 ▶).
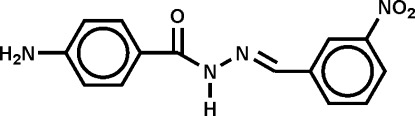


## Experimental

### 

#### Crystal data

C_14_H_12_N_4_O_3_
*M* *_r_* = 284.28
Monoclinic, 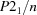

*a* = 7.8909 (16) Å
*b* = 11.153 (2) Å
*c* = 14.709 (3) Å
β = 92.00 (3)°
*V* = 1293.7 (4) Å^3^
*Z* = 4
Mo *K*α radiation
μ = 0.11 mm^−1^
*T* = 296 K
0.20 × 0.15 × 0.12 mm

#### Data collection

Bruker SMART CCD diffractometer
Absorption correction: multi-scan (*SADABS*; Sheldrick, 1996 ▶) *T*_min_ = 0.981, *T*_max_ = 0.987
11091 measured reflections
2952 independent reflections
2756 reflections with *I* > 2σ(*I*)
*R*_int_ = 0.044

#### Refinement

*R*[*F* ^2^ > 2σ(*F* ^2^)] = 0.057
*wR*(*F* ^2^) = 0.161
*S* = 1.06
2952 reflections
190 parameters
H-atom parameters constrained
Δρ_max_ = 0.22 e Å^−3^
Δρ_min_ = −0.22 e Å^−3^

### 

Data collection: *SMART* (Bruker, 2007 ▶); cell refinement: *SAINT* (Bruker, 2007 ▶); data reduction: *SAINT*; program(s) used to solve structure: *SHELXS97* (Sheldrick, 2008 ▶); program(s) used to refine structure: *SHELXL97* (Sheldrick, 2008 ▶); molecular graphics: *SHELXTL* (Sheldrick, 2008 ▶); software used to prepare material for publication: *SHELXTL*.

## Supplementary Material

Crystal structure: contains datablock(s) global, I. DOI: 10.1107/S1600536812014110/hb6724sup1.cif

Structure factors: contains datablock(s) I. DOI: 10.1107/S1600536812014110/hb6724Isup2.hkl

Supplementary material file. DOI: 10.1107/S1600536812014110/hb6724Isup3.cml

Additional supplementary materials:  crystallographic information; 3D view; checkCIF report

## Figures and Tables

**Table 1:** Hydrogen-bond geometry (Å, °)

*D*—H⋯*A*	*D*—H	H⋯*A*	*D*⋯*A*	*D*—H⋯*A*
N2—H2⋯N1^i^	0.86	2.47	3.183 (2)	141
N2—H2⋯O3^ii^	0.86	2.44	3.041 (2)	127
N1—H1*A*⋯O1^iii^	0.89	2.27	3.106 (2)	156

Symmetry codes: (i) 
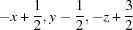
; (ii) 
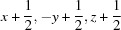
; (iii) 
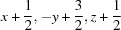
.

**Fig. 1.**
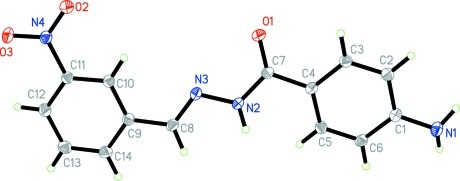
The molecular structure of the title compound, with displacement ellipsoids at the 30% probability level.

**Fig. 2.**
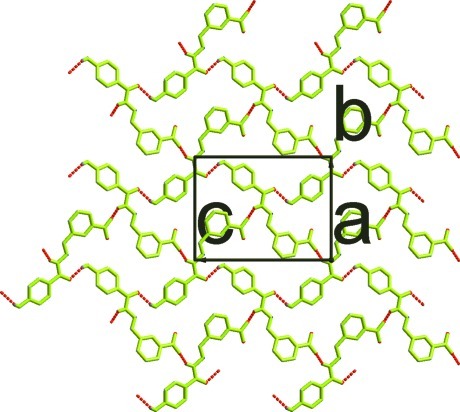
Crystal packing in the title compound where molecules are linked via N–H···O hydrogen bonds (dashed lines). Except for those involved in hydrogen-bonding interactions, H atoms have been omitted for clarity.

## supplementary crystallographic information

### Comment

As an extension of recent studies of hydrazone Schiff bases, (Cao, 2009; Zhou & Yang, 2010), the title compound was prepared and characterized.

As shown in Fig. 1, the asymmetric unit of the title compound, (I), contains one independent molecule displaying an *E* configuration with respect to its C═N double bond. The dihedral angle between the two benzene rings is 7.6 (4) °. The bond lengths and angles are as expected for a compound of this type and agree with the other ligands belonging to the hydrazone series. The C8═N3 and C7═O1 bond lengths of 1.283 (2) and 1.228 (2) Å, respectively, conform to the values for double bonds. Whereas the C1-N1, C8-N3, C11-N4 and N2-N3 [1.385 (2), 1.360 (2), 1.466 (2) and 1.378 (2) Å, respectively] bond lengths correspond to a single bond. In the crystal packing, it is noted that one of amino H (H1A) and amide H2 are involved in forming intermolecular N—H···O and N—H···N hydrogen bonds, which link the molecules into a R^4^_4_(36) graph-set notation (Fig. 2 and Table 1). These rings form an alternating sequence, in turn, linking the molecules into a two-dimensional supramolecular sheet structure parallel to (101). Neighboring layers are further interacting with each other through weak π–π stacking interactions [centroid to centroid distances of the benzene C1/C6 and C9/C14 rings are 3.93 (6) and 4.07 (6) Å].

### Experimental

3-Nitrobenzaldehyde (1 mmol, 0.151 g) and 4-aminobenzohydrazide (1 mmol, 0.151 g) were dissolved in MeOH (20 ml). The mixture was stirred for 6 hours at room temperature to give a yellow solution. Yellow prisms were formed by gradual evaporation of the solvent over a period of 5 days at room temperature.

### Refinement

H-atoms were placed in calculated positions (C—H = 0.93 and N—H = 0.86-0.89 Å) and were included in the refinement in the riding model approximation, with *U*_iso_(H) set to 1.2*U*_eq_(C or N).

### Crystal data

**Table:**

C_14_H_12_N_4_O_3_	*F*(000) = 592
*M**_r_* = 284.28	*D*_x_ = 1.460 Mg m^−^^3^
Monoclinic, *P*2_1_/*n*	Mo *K*α radiation, λ = 0.71073 Å
Hall symbol: -P 2yn	Cell parameters from 3352 reflections
*a* = 7.8909 (16) Å	θ = 1.4–27.5°
*b* = 11.153 (2) Å	µ = 0.11 mm^−^^1^
*c* = 14.709 (3) Å	*T* = 296 K
β = 92.00 (3)°	Prism, yellow
*V* = 1293.7 (4) Å^3^	0.20 × 0.15 × 0.12 mm
*Z* = 4	

### Data collection

**Table:**

Bruker SMART CCD diffractometer	2952 independent reflections
Radiation source: fine-focus sealed tube	2756 reflections with *I* > 2σ(*I*)
Graphite monochromator	*R*_int_ = 0.044
φ and ω scans	θ_max_ = 27.5°, θ_min_ = 2.8°
Absorption correction: multi-scan (*SADABS*; Sheldrick, 1996)	*h* = −10→10
*T*_min_ = 0.981, *T*_max_ = 0.987	*k* = −14→14
11091 measured reflections	*l* = −19→18

### Refinement

**Table:**

Refinement on *F*^2^	Primary atom site location: structure-invariant direct methods
Least-squares matrix: full	Secondary atom site location: difference Fourier map
*R*[*F*^2^ > 2σ(*F*^2^)] = 0.057	Hydrogen site location: inferred from neighbouring sites
*wR*(*F*^2^) = 0.161	H-atom parameters constrained
*S* = 1.06	*w* = 1/[σ^2^(*F*_o_^2^) + (0.0851*P*)^2^ + 0.6295*P*] where *P* = (*F*_o_^2^ + 2*F*_c_^2^)/3
2952 reflections	(Δ/σ)_max_ < 0.001
190 parameters	Δρ_max_ = 0.22 e Å^−^^3^
0 restraints	Δρ_min_ = −0.22 e Å^−^^3^

### Special details

**Table:**

Geometry. All esds (except the esd in the dihedral angle between two l.s. planes) are estimated using the full covariance matrix. The cell esds are taken into account individually in the estimation of esds in distances, angles and torsion angles; correlations between esds in cell parameters are only used when they are defined by crystal symmetry. An approximate (isotropic) treatment of cell esds is used for estimating esds involving l.s. planes.
Refinement. Refinement of F^2^ against ALL reflections. The weighted R-factor wR and goodness of fit S are based on F^2^, conventional R-factors R are based on F, with F set to zero for negative F^2^. The threshold expression of F^2^ > 2sigma(F^2^) is used only for calculating R-factors(gt) etc. and is not relevant to the choice of reflections for refinement. R-factors based on F^2^ are statistically about twice as large as those based on F, and R- factors based on ALL data will be even larger.

### Fractional atomic coordinates and isotropic or equivalent isotropic displacement parameters (Å^2^)

**Table:**

	*x*	*y*	*z*	*U*_iso_*/*U*_eq_	
O1	0.13734 (17)	0.68729 (12)	0.42167 (9)	0.0318 (3)	
O2	−0.0202 (2)	0.28779 (13)	0.14494 (10)	0.0405 (4)	
O3	0.00112 (17)	0.09872 (13)	0.11390 (9)	0.0352 (4)	
N1	0.3895 (2)	0.95096 (15)	0.79084 (11)	0.0333 (4)	
H1B	0.3783	1.0286	0.7780	0.040*	
H1A	0.4796	0.9306	0.8255	0.040*	
N2	0.2130 (2)	0.52763 (13)	0.50999 (10)	0.0280 (4)	
H2	0.2400	0.5032	0.5640	0.034*	
N3	0.18734 (19)	0.44493 (14)	0.44124 (10)	0.0270 (3)	
N4	0.02597 (19)	0.18540 (14)	0.16438 (10)	0.0286 (4)	
C1	0.3493 (2)	0.87791 (17)	0.71707 (12)	0.0266 (4)	
C2	0.2528 (2)	0.92102 (17)	0.64211 (12)	0.0294 (4)	
H2A	0.2201	1.0011	0.6402	0.035*	
C3	0.2060 (2)	0.84555 (16)	0.57111 (12)	0.0278 (4)	
H3	0.1413	0.8756	0.5222	0.033*	
C4	0.2540 (2)	0.72489 (16)	0.57130 (12)	0.0253 (4)	
C5	0.3551 (2)	0.68367 (17)	0.64523 (12)	0.0286 (4)	
H5	0.3921	0.6045	0.6459	0.034*	
C6	0.4007 (2)	0.75809 (17)	0.71689 (12)	0.0289 (4)	
H6	0.4663	0.7282	0.7655	0.035*	
C7	0.1965 (2)	0.64721 (16)	0.49378 (12)	0.0259 (4)	
C8	0.2188 (2)	0.33609 (17)	0.46441 (12)	0.0288 (4)	
H8	0.2524	0.3187	0.5242	0.035*	
C9	0.2025 (2)	0.23928 (16)	0.39805 (12)	0.0272 (4)	
C10	0.1297 (2)	0.25889 (16)	0.31199 (12)	0.0262 (4)	
H10	0.0924	0.3350	0.2949	0.031*	
C11	0.1133 (2)	0.16422 (16)	0.25250 (12)	0.0259 (4)	
C12	0.1707 (2)	0.04973 (17)	0.27327 (13)	0.0317 (4)	
H12	0.1596	−0.0123	0.2312	0.038*	
C13	0.2455 (3)	0.03068 (17)	0.35892 (14)	0.0345 (4)	
H13	0.2862	−0.0450	0.3748	0.041*	
C14	0.2596 (2)	0.12410 (18)	0.42087 (13)	0.0320 (4)	
H14	0.3078	0.1100	0.4785	0.038*	

### Atomic displacement parameters (Å^2^)

**Table:**

	*U*^11^	*U*^22^	*U*^33^	*U*^12^	*U*^13^	*U*^23^
O1	0.0356 (7)	0.0343 (7)	0.0249 (7)	0.0026 (6)	−0.0065 (5)	0.0016 (5)
O2	0.0494 (9)	0.0321 (8)	0.0390 (8)	0.0013 (6)	−0.0126 (7)	0.0013 (6)
O3	0.0339 (7)	0.0356 (7)	0.0356 (7)	−0.0054 (6)	−0.0054 (6)	−0.0092 (6)
N1	0.0338 (9)	0.0346 (9)	0.0312 (8)	−0.0009 (7)	−0.0027 (7)	−0.0080 (7)
N2	0.0357 (8)	0.0278 (8)	0.0200 (7)	−0.0016 (6)	−0.0046 (6)	−0.0012 (6)
N3	0.0278 (8)	0.0290 (8)	0.0240 (7)	−0.0020 (6)	−0.0015 (6)	−0.0035 (6)
N4	0.0267 (8)	0.0298 (8)	0.0294 (8)	−0.0023 (6)	−0.0011 (6)	−0.0009 (6)
C1	0.0210 (8)	0.0326 (9)	0.0262 (9)	−0.0028 (7)	0.0021 (6)	−0.0045 (7)
C2	0.0314 (9)	0.0256 (8)	0.0312 (9)	−0.0025 (7)	0.0011 (7)	−0.0001 (7)
C3	0.0277 (9)	0.0288 (9)	0.0267 (9)	−0.0001 (7)	−0.0016 (7)	0.0021 (7)
C4	0.0237 (8)	0.0290 (9)	0.0233 (8)	−0.0012 (7)	0.0008 (6)	−0.0008 (7)
C5	0.0282 (9)	0.0270 (9)	0.0302 (9)	0.0004 (7)	−0.0031 (7)	−0.0019 (7)
C6	0.0263 (9)	0.0344 (10)	0.0255 (9)	0.0006 (7)	−0.0057 (7)	−0.0009 (7)
C7	0.0233 (8)	0.0302 (9)	0.0239 (8)	0.0002 (7)	−0.0008 (6)	0.0000 (7)
C8	0.0283 (9)	0.0320 (9)	0.0256 (9)	0.0004 (7)	−0.0043 (7)	−0.0011 (7)
C9	0.0248 (8)	0.0292 (9)	0.0274 (9)	−0.0001 (7)	−0.0011 (7)	−0.0008 (7)
C10	0.0233 (8)	0.0258 (8)	0.0294 (9)	−0.0009 (7)	0.0002 (7)	−0.0005 (7)
C11	0.0240 (8)	0.0282 (9)	0.0254 (9)	−0.0017 (7)	0.0001 (7)	−0.0002 (7)
C12	0.0343 (10)	0.0271 (9)	0.0337 (10)	0.0014 (8)	0.0025 (8)	−0.0030 (7)
C13	0.0377 (10)	0.0282 (9)	0.0376 (10)	0.0084 (8)	0.0013 (8)	0.0012 (8)
C14	0.0323 (10)	0.0344 (10)	0.0292 (9)	0.0046 (8)	−0.0017 (7)	0.0030 (7)

### Geometric parameters (Å, º)

**Table:**

O1—C7	1.228 (2)	C4—C5	1.403 (3)
O2—N4	1.229 (2)	C4—C7	1.490 (2)
O3—N4	1.231 (2)	C5—C6	1.380 (2)
N1—C1	1.385 (2)	C5—H5	0.9300
N1—H1B	0.8899	C6—H6	0.9300
N1—H1A	0.8900	C8—C9	1.458 (2)
N2—C7	1.360 (2)	C8—H8	0.9300
N2—N3	1.378 (2)	C9—C10	1.389 (3)
N2—H2	0.8600	C9—C14	1.398 (3)
N3—C8	1.283 (2)	C10—C11	1.375 (2)
N4—C11	1.466 (2)	C10—H10	0.9300
C1—C6	1.397 (3)	C11—C12	1.385 (3)
C1—C2	1.403 (3)	C12—C13	1.389 (3)
C2—C3	1.382 (3)	C12—H12	0.9300
C2—H2A	0.9300	C13—C14	1.386 (3)
C3—C4	1.398 (3)	C13—H13	0.9300
C3—H3	0.9300	C14—H14	0.9300
			
C1—N1—H1B	112.8	C5—C6—H6	119.7
C1—N1—H1A	117.1	C1—C6—H6	119.7
H1B—N1—H1A	116.1	O1—C7—N2	122.65 (17)
C7—N2—N3	121.12 (15)	O1—C7—C4	123.07 (17)
C7—N2—H2	119.4	N2—C7—C4	114.27 (15)
N3—N2—H2	119.4	N3—C8—C9	120.75 (17)
C8—N3—N2	114.60 (15)	N3—C8—H8	119.6
O2—N4—O3	123.35 (16)	C9—C8—H8	119.6
O2—N4—C11	118.80 (15)	C10—C9—C14	118.88 (17)
O3—N4—C11	117.84 (15)	C10—C9—C8	121.15 (17)
N1—C1—C6	120.38 (17)	C14—C9—C8	119.97 (16)
N1—C1—C2	121.16 (17)	C11—C10—C9	119.06 (17)
C6—C1—C2	118.44 (17)	C11—C10—H10	120.5
C3—C2—C1	120.58 (17)	C9—C10—H10	120.5
C3—C2—H2A	119.7	C10—C11—C12	123.03 (17)
C1—C2—H2A	119.7	C10—C11—N4	118.02 (16)
C2—C3—C4	121.31 (17)	C12—C11—N4	118.92 (16)
C2—C3—H3	119.3	C11—C12—C13	117.80 (17)
C4—C3—H3	119.3	C11—C12—H12	121.1
C3—C4—C5	117.62 (16)	C13—C12—H12	121.1
C3—C4—C7	118.86 (16)	C14—C13—C12	120.21 (18)
C5—C4—C7	123.52 (17)	C14—C13—H13	119.9
C6—C5—C4	121.40 (17)	C12—C13—H13	119.9
C6—C5—H5	119.3	C13—C14—C9	120.99 (18)
C4—C5—H5	119.3	C13—C14—H14	119.5
C5—C6—C1	120.60 (17)	C9—C14—H14	119.5
			
C7—N2—N3—C8	175.27 (16)	N2—N3—C8—C9	−178.03 (15)
N1—C1—C2—C3	−176.57 (17)	N3—C8—C9—C10	−9.4 (3)
C6—C1—C2—C3	1.8 (3)	N3—C8—C9—C14	171.08 (18)
C1—C2—C3—C4	−0.5 (3)	C14—C9—C10—C11	1.1 (3)
C2—C3—C4—C5	−1.5 (3)	C8—C9—C10—C11	−178.44 (16)
C2—C3—C4—C7	178.64 (16)	C9—C10—C11—C12	−1.9 (3)
C3—C4—C5—C6	2.3 (3)	C9—C10—C11—N4	176.03 (15)
C7—C4—C5—C6	−177.89 (17)	O2—N4—C11—C10	4.2 (2)
C4—C5—C6—C1	−1.0 (3)	O3—N4—C11—C10	−175.03 (16)
N1—C1—C6—C5	177.33 (17)	O2—N4—C11—C12	−177.78 (17)
C2—C1—C6—C5	−1.1 (3)	O3—N4—C11—C12	3.0 (2)
N3—N2—C7—O1	9.7 (3)	C10—C11—C12—C13	1.0 (3)
N3—N2—C7—C4	−171.05 (15)	N4—C11—C12—C13	−176.84 (16)
C3—C4—C7—O1	14.6 (3)	C11—C12—C13—C14	0.6 (3)
C5—C4—C7—O1	−165.29 (17)	C12—C13—C14—C9	−1.3 (3)
C3—C4—C7—N2	−164.70 (16)	C10—C9—C14—C13	0.5 (3)
C5—C4—C7—N2	15.5 (3)	C8—C9—C14—C13	180.00 (18)

### Hydrogen-bond geometry (Å, º)

**Table:**

*D*—H···*A*	*D*—H	H···*A*	*D*···*A*	*D*—H···*A*
N2—H2···N1^i^	0.86	2.47	3.183 (2)	141
N2—H2···O3^ii^	0.86	2.44	3.041 (2)	127
N1—H1*A*···O1^iii^	0.89	2.27	3.106 (2)	156

Symmetry codes: (i) −*x*+1/2, *y*−1/2, −*z*+3/2; (ii) *x*+1/2, −*y*+1/2, *z*+1/2; (iii) *x*+1/2, −*y*+3/2, *z*+1/2.
